# Children's Day at the Metropolitan

**Published:** 1914-01-03

**Authors:** 


### Children's Day at the Metropolitan.

THE arrangements for Christmas at the Metropolitan Hospital included very prettily decorated wards—one, as if for Winter, with robin redbreasts in snow, red lamp-shades which were embroidered with black cats, and red and white flowers on the tables and window sills. One of the patients in this ward presented the sister with a little dog, "Tubby," which was given a place of honour on one of the tables. The scheme of colour in the ward immediately above was yellow and white. This was carried out in lamp-shades and tulips, relieved by moss, mimosa, and smilax. There were three small Christmas-trees covered with toys. The dolls on the trees were dressed by the patients in this ward. There were also yellow fairy lamps and owls and miniature Christmas-trees on the window sills. A third ward was to represent Spring. The scheme of colour here was pink, which was carried out in the lamp-shades, and with tulips and pink geraniums. Here, again, there was a plentiful supply of moss and smilax. On the beds there were white draperies, and green and white fairy lamps gave the ward a charming appearance. In the children's ward pink was the principal colour. There were sprays of almond blossom all round the ward, and pink silk lamp-shades with mistletoe. The cots were draped with white muslin and pink ribbons. At the head of each cot was a fairy doll, and at the foot a Christmas stocking. The children themselves were dressed in white embroidery, with pink ribbons. Palms and white tulips formed the table decoration. The mother of one child brought crackers for the children, and another Christmas stockings. A member of the residential medical staff presented the ward with a hand cycle, which gave great pleasure to the little ones. There were also owl fairy lamps in various parts of the ward, which were very effective. In every instance the arrangements for the wards were made by the sister.

There are 123 patients in the Metropolitan Hospital, and after breakfast Father Christmas appeared—this year a house physician, Mr. B. W. Brewitt. He was accompanied by a clown, a suffragette, and other amusing characters taken by members of the resident staff. He presented every patient with a parcel, those for the men containing a shirt, necktie, and a pair of socks; and those for the women a petticoat, shawl, and other useful articles of clothing. The children fared well, as Father Christmas brought them many toys. These gifts were kindly provided by the Christmas Fairies, the Central Foundation School, the Santa Claus Society, Messrs. J. Rotherham, the Editor of *Truth* (who sent a large doll dressed as "Bunty"), Mrs. Weatherley, and the Ladies' Association.

Turkey and plum-pudding were provided for all the patients who were able to take them. These dinners were carved by the doctors and sisters in the wards. The Christmas pudding was decorated with holly, and carried round the ward flaming amid the cheers of the patients. On this day all the patients were allowed to smoke. Tobacco, cigarettes, and pipes were kindly provided by Mr. J. Dunnington Jefferson, Mr. A. Lister Harrison, and several firms. Visitors were allowed to see the patients in the afternoon, and every patient was allowed to keep one friend to tea and to spend the evening.

After a good tea (with fruit and cakes) and plenty of Christmas crackers provided by the Christmas Entertainment Fund, the evening's entertainment began. This included a variety entertainment by the resident medical staff, entitled "The Dear Departed"; a farce, "Miss Honey's Treasure," by the matron and home sister; a play, "Beauty and the Beast," by the night nurses; a Dutch concert troupe by the day nurses; and Mrs. Jarley's waxworks. The Misses Cressall also assisted with violin and piano. These performances were given in two of the surgical wards, and all medical patients who were able to be moved were taken to enjoy the fun.

